# Transforming growth factor-β1 and SMAD signalling pathway in the small airways of smokers and patients with COPD: potential role in driving fibrotic type-2 epithelial mesenchymal transition

**DOI:** 10.3389/fimmu.2023.1216506

**Published:** 2023-06-26

**Authors:** Samuel James Brake, Wenying Lu, Collin Chia, Greg Haug, Josie Larby, Ashutosh Hardikar, Gurpreet K. Singhera, Tillie L. Hackett, Mathew Suji Eapen, Sukhwinder Singh Sohal

**Affiliations:** ^1^ Respiratory Translational Research Group, Department of Laboratory Medicine, School of Health Sciences, College of Health and Medicine, University of Tasmania, Launceston, TAS, Australia; ^2^ Respiratory Medicine, Launceston Respiratory and Sleep Centre, Launceston, TAS, Australia; ^3^ Department of Respiratory Medicine, Launceston General Hospital, Launceston, TAS, Australia; ^4^ Department of Cardiothoracic Surgery, Royal Hobart Hospital, Hobart, TAS, Australia; ^5^ Department of Anaesthesiology, Pharmacology & Therapeutics, University of British Columbia, Vancouver, BC, Canada; ^6^ University of British Columbia (UBC) Centre for Heart Lung Innovation, St. Paul's Hospital, Vancouver, BC, Canada

**Keywords:** COPD - chronic obstructive pulmonary disease, TGF — transforming growth factor, epithelial to mesenchymal transition, smoking, small airway

## Abstract

**Background:**

COPD is a common disease characterized by respiratory airflow obstruction. TGF-β1 and SMAD pathway is believed to play a role in COPD pathogenesis by driving epithelial mesenchymal transition (EMT).

**Methods:**

We investigated TGF-β1 signalling and pSmad2/3 and Smad7 activity in resected small airway tissue from patients with; normal lung function and a smoking history (NLFS), current smokers and ex-smokers with COPD GOLD stage 1 and 2 (COPD-CS and COPD-ES) and compared these with normal non-smoking controls (NC). Using immunohistochemistry, we measured activity for these markers in the epithelium, basal epithelium, and reticular basement membrane (RBM). Tissue was also stained for EMT markers E-cadherin, S100A4 and vimentin.

**Results:**

The Staining of pSMAD2/3 was significantly increased in the epithelium, and RBM of all COPD groups compared to NC (p <0.0005). There was a less significant increase in COPD-ES basal cell numbers compared to NC (p= 0.02). SMAD7 staining showed a similar pattern (p <0.0001). All COPD group levels of TGF-β1 in the epithelium, basal cells, and RBM cells were significantly lower than NC (p <0.0001). Ratio analysis showed a disproportionate increase in SMAD7 levels compared to pSMAD2/3 in NLFS, COPD-CS and COPD-ES. pSMAD negatively correlated with small airway calibre (FEF_25–75%_; p= 0.03 r= -0.36). EMT markers were active in the small airway epithelium of all the pathological groups compared to patients with COPD.

**Conclusion:**

Activation of the SMAD pathway via pSMAD2/3 is triggered by smoking and active in patients with mild to moderate COPD. These changes correlated to decline in lung function. Activation of the SMADs in the small airways is independent of TGF-β1, suggesting factors other than TGF-β1 are driving these pathways. These factors may have implications for small airway pathology in smokers and COPD through the process of EMT, however more mechanistic work is needed to prove these correlations.

## 1 Introduction

Chronic Obstructive Pulmonary Disease (COPD) is a common but preventable chronic lung disease (1, 2). COPD is the third leading cause of death worldwide (3, 4). COPD is caused by obstruction and limitations to the respiratory airflow (3–5). These airflow limitations primarily arise from the reduction of the luminal diameter of the bronchioles and destruction of the parenchymal alveolar tissue. The most significant risk factor for COPD is cigarette smoking; however, air pollution and fossil fuel exposure are also recognized risks (6). Prolonged vaping and long-term use of electronic cigarettes may also lead to the development and progression of COPD, but given the infancy of these products, the clinical presentation of eCig-driven COPD is yet to emerge (7, 8). Chronic insult from cigarette smoke and/or air pollutants results in progressive structural changes in both the large and small airways and lung parenchyma, resulting in significant airflow limitation of the lung due to increased airway wall thickness (1, 9, 10). Increased airway wall thickening results in part from pro-inflammatory changes, such as inflammatory cell infiltration, but also from structural changes, such as mucous gland hyperplasia, reticular basement membrane thickening and fragmentation, vascular proliferation and smooth muscle hypertrophy (11–15). The relationship between airway thickness and the pathophysiology of COPD was demonstrated by a study showing an inverse relationship between airway wall thickening and the degree of airflow obstruction, which is positively related to a cumulative smoking history (16). Remodelling in the sub-epithelium leads to small airway narrowing and rigidity. It is considered the most important contributor to an accelerated decline of FEV_1_, a clinical test of pulmonary function (9, 10, 17). At the core of these remodeling events in COPD is the epithelial-mesenchymal transition (EMT) (1, 18–20).

In response to pro-inflammatory and pro-fibrotic mediators, polarized epithelial cells are known to undergo the transition from an epithelial to a mesenchymal phenotype (9, 21, 22). This phenotypic change includes increased cellular migratory capacity and associated invasive properties, resistance to apoptosis and increased abnormal deposition of extracellular matrix (ECM), ultimately increasing airway wall thickness (21). Transitioning cells migrate through the reticular basement membrane (RBM) by degrading the underlying membrane. The transformed cells are likely to then migrate away from the epithelium and lodge themselves in the underlying tissue towards the inner mucosa (21–23).

There are two types of pathological EMT processes involved in COPD. Type 2 EMT is seen in small airways, giving rise to myofibroblasts from epithelia to heal injured tissues. In ongoing chronic assault, from cigarette smoking, for example, abnormal formation of myofibroblasts cause progressive fibrosis, thereafter leading to lung parenchymal destruction and small airway remodeling (24). Type 3 EMT is active in the large airways and provides a critical link between smoking and a pro-cancer phenotype (25).

It has been previously shown that Type 3 EMT in the large airways of smokers and COPD patients is driven by the transforming growth factor beta one (TGF-β1) and activated small mothers against the decapentaplegic (SMAD) pathway (26). TGF-β is a family of multifunctional cytokines (TGF-β_1_, TGF-β_2_, and TGF-β_3_). They are known to regulate cellular proliferation and differentiation, apoptosis, angiogenesis, and ECM synthesis. TGF-β1 play a significant role in COPD pathogenesis through their pleiotropic chemoattraction properties targeting several cell types, including monocytes and neutrophil, and by recruiting and inducing fibroblasts, possibly through the EMT process (26–28).

TGF-β acts through TGF-β receptors (TGF-βRs) I, II, and are known to feed into both the “canonical” and “non-canonical” pathways. TGF-β1 action via the canonical cascade involves the phosphorylation of SMAD transcription factors (29). Binding of TGF-β1 to TGF-βR II upregulates the genetic expression of EMT related genes via the SMAD pathway (30). TGF-β1 binding to TGF-βR II phosphorylates SMAD2/3 (27). This results in a conformational change, forming a receptor complex with SMAD4, and allowing translocation to the nucleus. The pSMAD complex binds to a promotor on the target EMT genes, activating genetic expression (26, 27). SMAD7 is an inhibitory SMAD that downregulates TGF-β signalling through stable binding to the TGF-β1 receptor complex, competitively inhibiting the binding of SMAD2/3 (27).

Mahmood et al. from our group previously showed an increase in TGF-β1 levels in large airways of normal lung function smokers (NLFS) compared to non-smoking normal controls (NC), which was further exacerbated in COPD current smokers (COPD-CS). This elevation of TGF-β1 in the large airways has been identified as a likely driver of EMT type 3 (18, 31). In the large airways, the TGF-β1 expression of ex-smoking COPD patients has been shown to return to levels comparable to normal lung function smokers. This suggests a partially reversible process (26). The same trend was observed in pSMAD 2/3 and an inverse trend of inhibitory SMAD 7 (26). We now further investigate the TGF‐β1 and SMAD levels in the small airways of patients with stable COPD, NLFS and NC.

## 2 Materials and methods

### 2.1 Subjects

Surgically resected lung tissue was available from our biobank. Tissue was taken from thirty-nine individuals; eleven normal lung function smokers (NLFS), nine current smokers with COPD (COPD-CS) and eight COPD ex-smokers (COPD-ES) and compared with eleven non-smoking normal controls (NC) which were deceased other than respiratory diseases (**Table 1**). The surgically resected material was taken well away from any primary tumour and contained non-cancer-affected small airways. The surgeries were performed by the thoracic surgeon, co-author on this paper (Hardikar A). COPD was diagnosed according to the Global Initiative for Chronic Obstructive Lung Disease (GOLD) criteria, and the subjects used in this study had mild to moderate COPD, with the majority in the mild category (airflow limitation of FEV1 ≥ 80 % predicted). Subjects with other respiratory diseases and a history of recent acute exacerbation, or those on systemic or inhaled corticosteroids were excluded from the study. Ethics approval for the study was approved by the Tasmania Health & Medical Human Research Ethics Committee approved the study (H0012374). The non-smoking normal control tissues were obtained through the James Hogg Lung Registry, the University of British Columbia, with approval from the Providence Health Care Research Ethics Board H00–50110. All subjects gave prior written, informed consent.

**Table 1:** Demographic details and lung function data for participants.

Groups	NC	NLFS	COPD-CS	COPD-ES
Subjects (n)	11	11	9	8
Age (years)	68 (63–75)	70 (52–79)	64 (59–78)	68 (56–85)
Smoking (Pack/year)	0	33 (3–60)	28.5 (2–50)	33 (18–36)
FEV_1_/FVC (post BD)†	N/A	76 (70–90)	66 (60–70)	64 (55–69)

Data represented here as median and range in brackets.

NC- Normal non-smoker controls, NLFS- Normal Lung Function Smokers, COPD-CS- COPD-Current Smokers and COPD-ES- COPD-Ex Smoker.

FEV1/FVC- Ratio of the forced expiratory volume in the first one second to the forced vital capacity of the lungs.

†Post BD values after 400 µg of salbutamol.

### 2.2 Immunohistochemistry

Tissue was fixed in formalin before processing. Paraffin-embedded sections were cut at 3-4 µm for staining on charged slides. After deparaffinisation and heat induced epitope retrieval (HIER), sections were treated with a 15-minute 3% hydrogen peroxide endogenous enzyme block (216763-500ML, Merck, Darmstadt, Germany) before applying the primary antibody. At ambient temperature, sections were then stained with: monoclonal antibody anti-TGF-β1 (27969 clone TB1 Clone TB21, Abcam, Cambridge, UK) at 1:4000 dilution for 90 minutes after a 30-minute protein block (X090930-2, Dako, Victoria, Australia), phosphorylated pSMAD 2/3 (SC-11769r, santa-Cruz, California, USA) at 1:100 dilution for 60 minutes, SMAD 7 (Santa Cruz SC-101152, California, USA) at 1:50 dilution for 60 minutes, E-cadherin (M3612, Dako, Victoria, Australia) at 1:50 dilution for 60 minutes, mouse monoclonal Vimentin (M7020 Dako, Victoria, Australia) at 1:200 dilution for 60 minutes, and rabbit polyclonal S100A4 (A5114, Dako, Victoria, Australia) at 1:1000 dilution for 60 minutes after a 30-minuts protein block (X090930-2, Dako, Victoria, Australia) respectively. Bound primary antibodies were visualised by the Envision+ system, utilising rabbit/mouse targeting secondary antibodies (K406311-2, DAKO, Victoria, Australia), horseradish peroxidase and diaminobenzidine (DAB) for a brown colour resolution (K5007, Dako, Victoria, Australia). Gills type II haematoxylin (AHG2.1L, Australian Biostain, Victoria, Australia) was applied as a nuclear counterstained, followed by bluing in ammoniated water (221228-1L-A, Sigma-Aldrich, Victoria, Australia). Sections were dehydrated through ascending grades of ethanol, cleared in xylene and mounted in CV mount (14046430011, Leica, Illinois, USA).

### 2.3 Tissue section quantification and analysis

All slides were coded and randomised to blind the analyst (S.J.B.). Images of the small airway epithelium (small airways defined as less than 2 mm in diameter and lacking cartilaginous support) were taken, and overlapping was strictly avoided. Eight images were randomly selected from the total images using an online randomisation generator for measurements from each slide, for each of the biomarkers. Images were taken using a Lecia ICC50 W camera mounted to a Lecia DM 500 microscope at 40x magnification at the bright field. Measurements were performed by computer-assisted image analysis using Image Pro Plus V7.0 software (Media Cybernetics, USA). All biomarkers (TGF-β1, pSMAD 2/3 and SMAD 7) were quantified as percentage epithelial area showing positive staining and number of positively stained basal epithelial cells (identified as small, nearly cuboidal cells with prominent nuclei, attached to the basement membrane, and differentiated from pseudostratified, or simple epithelia based on histological differences) and cells in the RBM per mm of RBM length.

### 2.4 Statistical analysis

Following a normal distribution check of each data set, the results for each quantification were presented as mean and standard error of difference. One‐way analysis of variance (ANOVA) parametric tests (Tukey, comparing means across groups) were applied. Statistical analyses were performed using GraphPad Prism v8.0. Linear regression and Spearman r’ were used for correlation analysis. A p-value of ≤0.05 was considered statistically significant for all group comparisons and correlations.

## 3 Results

### 3.1 TGF-β1 is suppressed in the small airway epithelium and reticular basement membrane of smokers and COPD patients

#### 3.1.1 Epithelium

**Figure 1:**
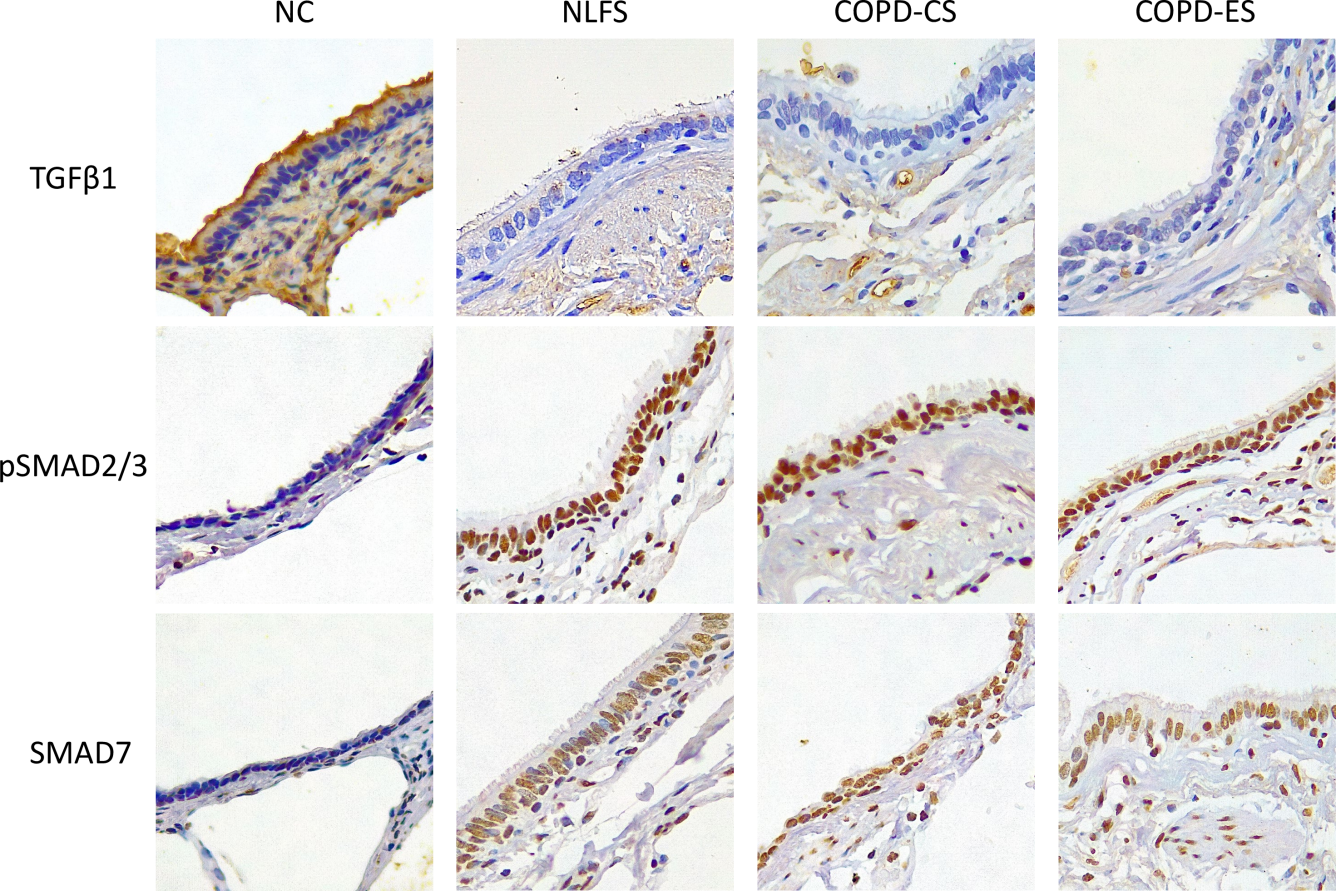
Micrographs showing immunohistochemical staining for biomarker TGF-β1, pSMAD2/3 and SMAD7 for NC, Normal non-smoker controls; NLFS, Normal Lung Function Smokers; COPD-CS, COPD Current Smokers and COPD-ES, COPD Ex-Smoker. Original magnifications, 40x. Scale bar = 10µm.

**Figure 2:**
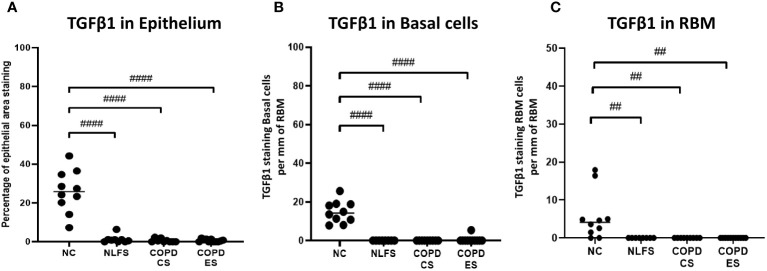
The TGF- β1 immunohistochemical staining expression in small airways across all groups. Positive TGF- β1 is expressed as **(A)** percentage of bronchiolar epithelium, **(B)** number of basal cells per mm of reticular basement membrane, and **(C)** number of cells in the reticular basement membrane per mm of reticular basement membrane. Significance is indicated as ^##^p< 0.01 and ^####^p<0.0001. Tukey’s multiple comparison test was used for statistical analysis.

It was noted that almost no positive staining was seen in the NLFS and COPD samples (**Figure 1**). The NC epithelial cells stained strongly for TGF-β1 on the apical surface and somewhat in the cytoplasm but showed minimal nuclear staining. In NLFS, the most intense area of positive staining was on the apical border of the nucleus. The normal subjects had 26% of epithelium positively stained for TGF-β1. This was significantly greater than the NLFS and COPD groups which showed almost complete suppression of TGF-β1 in mild-moderate COPD and NLFS compared to NC (NLFS: 1% positive staining, COPD-CS: 0.6% and COPD-ES: 0.6%) (**Figures 1**, **2**, **Table 2**).

**Table 2:** Immunohistochemical quantification of TGF-β signalling pathway in small airway samples.

	NC mean	NLFS	COPD-CS	COPD-ES
TGF-β1				
Epithelium	26.05	24.84 (2.58) ^****^	25.46 (2.58) ^****^	25.44 (2.46) ^****^
Basal Cells	14.79	14.79 (1.44) ^****^	14.79 (1.39) ^****^	14.31 (1.32) ^****^
RBM Cells	5.65	5.65 (1.55) ^**^	5.65 (1.50) ^**^	5.65 (1.43) ^**^
pSMAD2/3				
Epithelium	10.13	- 21.24 (4.70) ^***^	- 26.84 (4.55) ^****^	- 32.91 (4.33) ^****^
Basal Cells	27.04	- 35.77 (5.64) ^**** ##^	- 43.97 (5.46) ^**** ####^	- 16.00 (5.19) *
RBM Cells	5.72	- 22.69 (3.62) ^****^	- 25.12 (3.50) ^****^	- 24.79 (3.33) ^****^
SMAD7				
Epithelium	1.278	- 15.13 (2.85) ^****^	- 11.16 (2.85) ^**^	- 14.02 (2.62) ^****^
Basal Cells	5.147	-28.11 (5.63) ^***^	-19.44 (5.63) ^**^	-23.52 (5.18) ^***^
RBM Cells	3.221	-23.95 (5.22) ^***^	-18.07 (5.22) ^**^	-19.77 (4.81) ^**^

Data are expressed as mean difference [NC mean - test group mean] (standard error of difference). Epithelium denotes the percentage of the area of epithelium showing positive staining. Basal Cells denote the number of basal epithelial cells staining positive per mm of RBM. RBM Cells denote the number of cells in the RBM staining positive per mm of RBM. Significance of the difference between test samples and NC is indicated as *p <.05, **p< 0.01, ***p<0.001 and ****p<0.0001. Significance of the difference between test samples and ES-COPD is indicated as ##p< 0.01 and ####p<0.0001 the Tukey’s multiple comparison test was used for statistical analysis. NC, Normal non-smoker controls; NLFS, Normal Lung Function Smokers; COPD-CS, COPD Current Smokers and COPD-ES, COPD Ex-Smoke; RBM, Reticular basement membrane.

#### 3.1.2 Basal cells

Compared to normal controls with 15 basal cells per mm of RBM staining positive for TGF-β1, there was a significant decrease in staining for TGF-β1 in basal cells from NLFS (0.0 cells showing positive staining per mm of RBM) and COPD patients(0.5 cells showing positive staining per mm of RBM, **Figures 1**, **2**, **Table 2**).

#### 3.1.3 RBM cells

The normal subjects had 6 cells in the RBM per mm of RBM staining positive for TGF-β1. This was significantly greater than the NLFS and COPD groups which showed complete suppression of TGF-β1 in RBM cells, with no positive staining reported in these groups (All pathological groups: 0.0 cells showing positive staining per mm RBM) (**Figures 1**, **2**, **Table 2**).

### 3.2 Increased expression of pSMAD2/3 in the epithelium and RBM of small airways in smokers and COPD patients

#### 3.2.1 Epithelium

**Figure 3:**
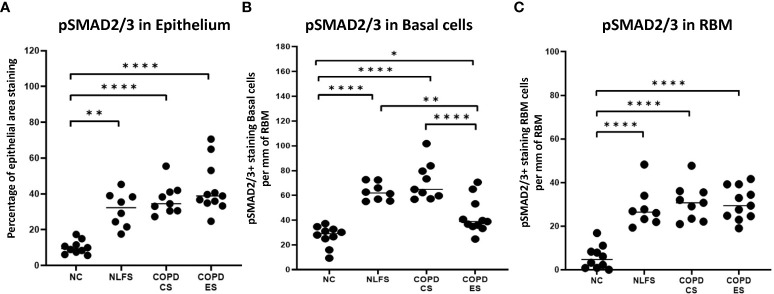
The pSMAD2/3 immunohistochemical staining expression in small airways across all groups. Positive pSMAD2/3 is expressed as **(A)** percentage of bronchiolar epithelium, **(B)** number of basal cells per mm of reticular basement membrane, and **(C)** number of cells in the reticular basement membrane per mm of reticular basement membrane. Significance is indicated as *p <.05, **p< 0.01 and ****p<0.0001. Tukey’s multiple comparison test was used for statistical analysis.

It was noted that in all NLFS and COPD groups, the location of most intense staining in all areas analysed (epithelium, basal cells and RBM) was the nucleus (**Figure 1**), while epithelial cells from NC showed positive staining most concentrated in the cilia and apical cytosol with any nuclear staining seen predominately in basal cells (**Figure 1**). The normal subjects had 10% of epithelium staining positive for pSMAD2/3. There was a significantly increased expression of pSMAD2/3 in the epithelium for all NLFS and COPD groups compared to NC (**Figure 3**). This increased expression was sustained in samples from COPD-ES (NLFS: 31% positive staining, COPD-CS: 37% and COPD-ES: 43%). There was no significant difference between NLFS and the COPD groups in expression of pSMAD2/3 in the epithelium and RBM (**Figures 1**, **3**, **Table 2**).

#### 3.2.2 Basal cells

Normal subjects showed 27 cells per mm of RBM staining positive. Expression of pSMAD2/3 significantly increased upon smoking stimulus in the NLFS and COPD-CS groups in basal epithelial cells (63 and 71 cells per mm of RBM staining positive, respectively) (**Figure 3**). There was a significant decrease in pSMAD2/3 positive basal epithelial cells per mm of RBM in COPD-ES compared to both NLFS and COPD-CS groups (COPD-ES: 43 cells per mm of RBM staining positive). While a reduction of pSMAD2/3 was seen in COPD-ES compared to NLFS and COPD-CS, basal cell levels in COPD-ES were still elevated to significant levels above those of the normal controls (**Figures 1**, **3**, **Table 2**).

#### 3.2.3 RBM cells

The numbers of cell staining positively for pSMAD2/3 in the RBM showed increases between all test groups compared to the NC. Normal subjects showed 6 cells per mm of RBM staining positive, at least 28 cells per mm of RBM staining positive were observed in NLFS and COPD groups (**Figures 1**, **3**, **Table 2**).

### 3.3 Increase in regulatory SMAD7 in the small airways of smokers and COPD patients

#### 3.3.1 Epithelium

**Figure 4:**
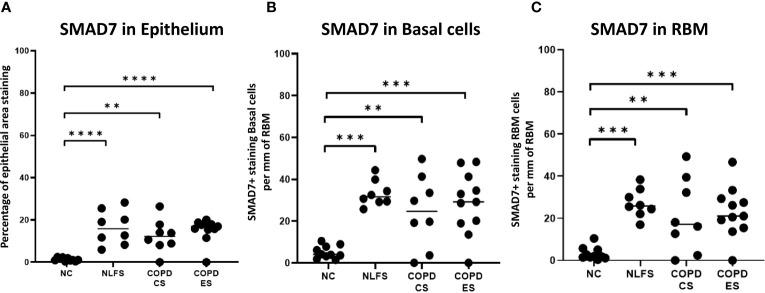
The SMAD7 immunohistochemical staining expression in small airways across all groups. Positive SMAD7 is expressed as **(A)** the percentage of bronchiolar epithelium, **(B)** number of basal cells per mm of reticular basement membrane, and **(C)** number of cells in the reticular basement membrane per mm of reticular basement membrane. Significance is indicated as **p< 0.01, ***p<0.001 and ****p<0.0001. Tukey’s multiple comparison test was used for statistical analysis.

As with pSMAD2/3, the area of most intense staining for SMAD7 in all areas analysed (epithelium, basal cells and RBM) was seen in the nucleus (**Figure 1**). SMAD7 significantly increased in the epithelium (**Figure 1**) of all test groups compared to the NC. An average of 1% epithelial staining positive for SMAD7 was observed in normal controls. An increase of at least 11% of epithelial staining was observed in all NLFS and COPD groups (**Figure 4**). There was no observed significant difference between NLFS and the COPD groups (**Figures 1**, **4**, **Table 2**).

#### 3.3.2 Basal cells

A significant increase of SMAD7 expression upon smoking stimulus was also observed in the basal epithelial cells (**Figure 1**). The number of basal cells staining positive per mm of RMB observed in normal subjects was 5. This increased significantly for all NLFS and COPD groups, with no significant differences observed between the pathological groups (NLFS: 33 cells per mm of RBM staining positive; COPD-CS: 25 cells per mm of RBM staining positive; and COPD-ES: 29 cells per mm of RBM staining positive) (**Figures 1**, **4**, **Table 2**).

#### 3.3.3 RBM cells

The number of cells stained positively for SMAD7 in the RBM showed highly significant increases between all test groups compared to the normal controls. Normal subjects showed 3 cells per mm of RBM staining positive in the RBM. This increased significantly in all NLFS and COPD groups, with no significant differences observed between the pathological groups (NLFS: 27 cells per mm of RBM staining positive, COPD-CS: 21 cells per mm of RBM staining positive and COPD-ES: 23 cells per mm of RBM staining positive) (**Figures 1**, **4**, **Table 2**).

### 3.4 Disproportionate increase of regulatory SMAD7 compared to pSMAD2/3

**Figure 5:**
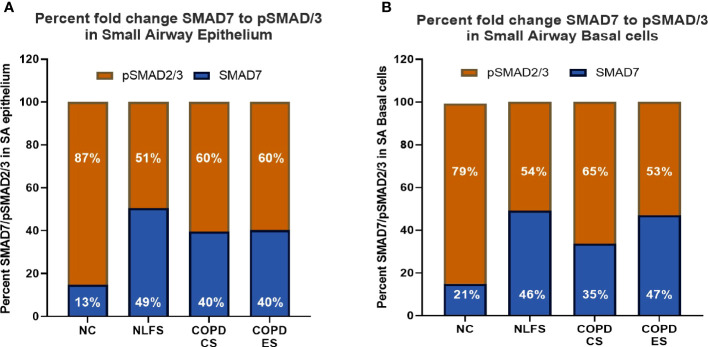
The ratio of positive expression of pSMAD2/3 to SMAD7 in small airways. **(A)** the ratio of positive pSMAD2/3 to SMAD7 staining in the epithelium is expressed as a percentage for all groups. **(B)** the ratio of positive pSMAD2/3 to SMAD7 staining basal cells per mm of reticular basement membrane is expressed as a percentage for all groups.

The ratio of pSMAD2/3 to SMAD7 in normal controls showed total SMAD staining split to 87% pSMAD2/3 to 13% SMAD 7, showing a significantly greater percentage of the epithelium that shows positive staining for either pSMAD2/3 or SMAD7, staining positive for pSMAD2/3. In normal controls only approximately 11% of the epithelium stain for both markers in total (**Figure 5**, **Table 2**). This increased to an approximate total SMAD split of 50% SMAD7, 50% pSMAD2/3 for NLFS, with almost 50% of the total epithelium staining positive. The proportions and total area staining decreased slightly in COPD-CS, and while the proportion remained the same in COPD-ES patients, the total epithelium staining positive for either marker increased to well over 50% (**Figure 5**, **Table 2**).

This ratio is similar observed in basal epithelial cells (**Figure 5**, **Table 2**). The ratio of pSMAD2/3 to SMAD7 in normal controls showed 79% pSMAD2/3 to 21% SMAD 7, with approximately 30 basal epithelial cells staining positive for either marker per mm of RBM (**Figure 5**, **Table 2**). This increased to 46% SMAD7, 54% SMAD2/3 for NLFS, with approximately 90 basal epithelial cells staining positive for either marker per mm of RMB. The proportions and number of cells decreased in COPD-CS. While the proportion of SMAD7 and pSMAD2/3 remained the same in COPD-ES as NLFS, the total number of basal cells staining decreased to less than 70 per mm of RBM (**Figure 5**, **Table 2**).

### 3.5 Correlation between biomarkers and lung function

**Figure 6:**
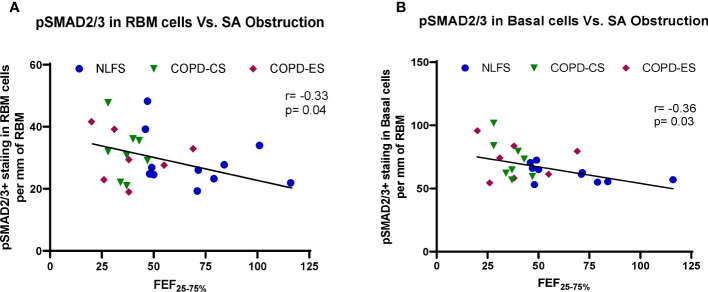
The correlation between pSMAD2/3 expression and small airway obstruction. **(A)** correlation of positive pSMAD2/3 staining in RBM cells and small airway obstruction marker FEF_25–75%_ across all subjects, and **(B)** correlation of positive pSMAD2/3 staining in basal cells and small airway obstruction marker FEF_25–75%_ across all subjects. FEF_25–75%_, Forced Expiratory Flow 25-75%.

Correlations between pSMAD levels and lung function suggested a general negative trend between the levels of pSMAD2/3 and lung function. Increased basal epithelial and RBM pSMAD2/3 cell expression was associated with decrease in small airway calibre (r= -0.33, p= 0.04 and r= -0.36, p= 0.03, respectively) (**Figure 6**). No correlation was found between SMAD7 and either lung function or small airway obstruction.

### 3.6 Descriptive analysis of EMT markers in small airway epithelium and RBM

**Figure 7:**
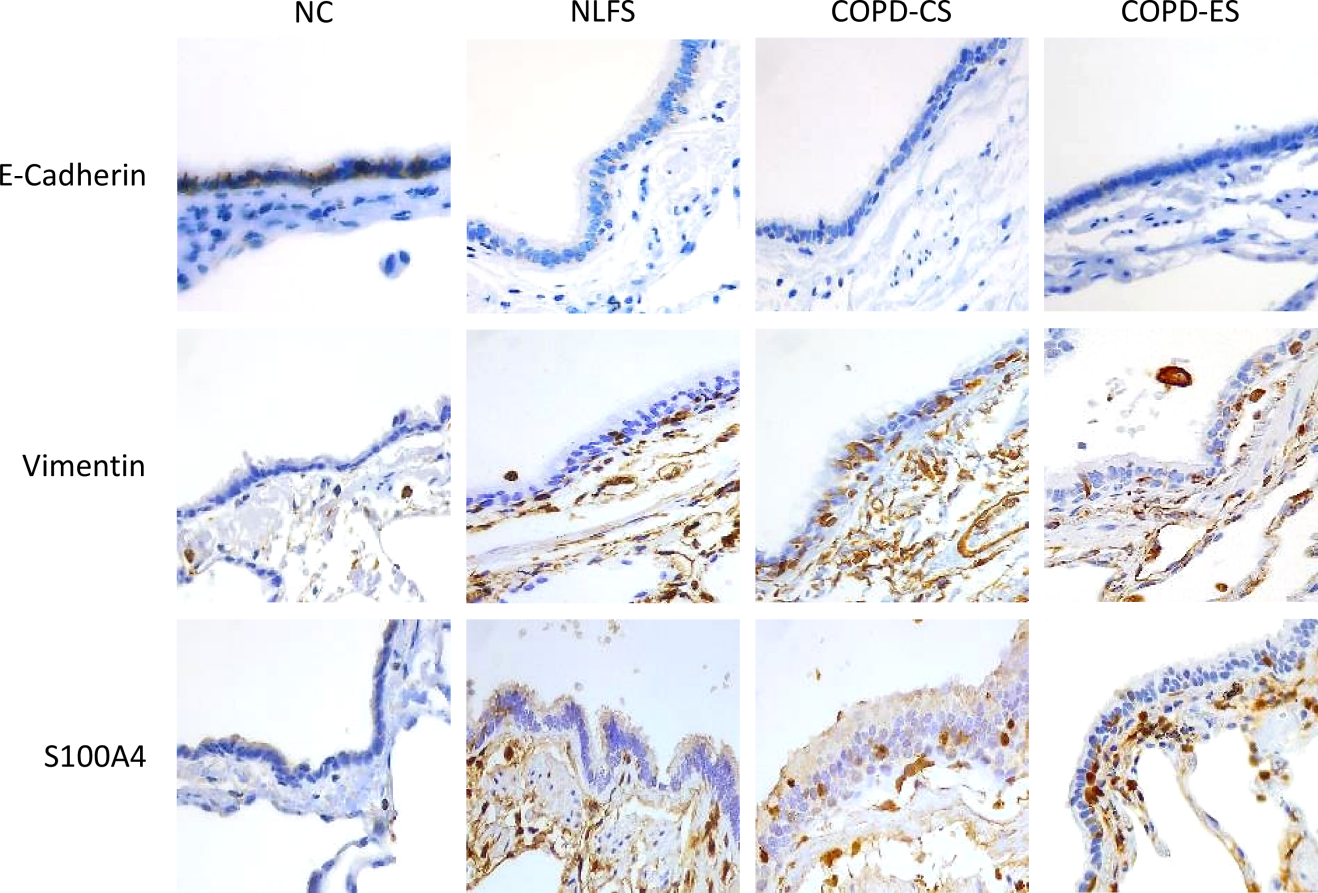
Representative images of EMT marker E-Cadherin, Vimentin, and S100A4 expression in the small airway across groups, NC, Normal non-smoker controls; NLFS, Normal Lung Function smokers; COPD-CS, COPD Current Smokers and COPD-ES, COPD Ex-Smokers. Original magnifications, 40x at the bright field.

We observed the increased expression of the mesenchymal markers, Vimentin and S100A4, in the small airway epithelium and RBM in the NLFS, COPD-CS and COPD-ES groups compared to the NC, the epithelial marker E-cadherin junctional expression was reduced in these groups compared to NC, indicating the epithelial cells transitioning to mesenchymal phenotype (**Figure 7**).

## 4 Discussion

This study demonstrates that TGF-β1 levels in the small airway tissue of smokers with and without COPD is significantly suppressed compared to normal controls (non-smokers and is associated with the activation of the SMAD pathway via pSMAD2/3. We also found active EMT in the same set of patients. The study shows this activation is triggered by smoking and also active in patients with mild to moderate COPD. We noted a paradoxical finding that the activation of the SMAD signalling pathway in the small airways occurred despite suppression of TGF-β1, and therefore is independent of TGF-β1 activation, suggesting factors other than TGF-β1 are driving these pathways. This finding in small airways is contrary to previous findings in the large airways, which showed an increase in TGF-β1 levels in large airways of normal lung function smokers (NLFS) compared to non-smoker controls (NC), which further aggravated in COPD current smokers. Increased pSMAD2/3 has been observed in the small airways of COPD patients, as demonstrated by this study, and in large airways, demonstrated by earlier studies (26). The disproportionate increase in SMAD7 observed in this study, however, suggests that the abhorrent SMAD pathway activation resulting in type 2 EMT, as seen in small airways, is mechanically different to those pathways previously established in EMT type 3 as seen in the large airways.

The early development of COPD is characterised by small airway destruction and fibrosis (32). While the cause of this is not well established, there is increasing evidence that basal epithelial cell dysregulation and transformation, through the action of EMT, is a driving factor (26). TGF-β has been shown to be a driving factor in the EMT pathway (33). Previous work published by our research group showed the likely importance of the TGF-β1-pSMAD pathway as a regulator of large airway EMT resulting in physiological obstruction and fibrosis. Findings demonstrated a positive association between smoking, COPD, and upregulation of stimulatory pSMADs (SMAD2/3), however, concluded that other factors to TGF-β1 were driving the process (18, 26). This study shows a dysregulation of the TGF-β canonical pathway resulting in increased activation of the SMAD signalling pathway independent of TGF-β1 activity in the small airways.

Increasing evidence supports dysregulated epithelial basal stem cell function as a feature active in EMT (34–36). TGF-β1 has been suggested as a key regulator of the fibrotic large airway disease in COPD (26, 37). There is currently a lack of consensus in support of this (27). This study showed epithelial levels of TGF-β1 in normal controls showed significantly higher positive epithelial staining than all pathological groups. This level of TGF-β1 in small airway epithelial tissue is supported by previous studies (27, 38), however other studies have shown an increase in TGF-β1 mRNA and protein in bronchiolar and alveolar epithelium in COPD patients, contradicting the findings of this study (39, 40). An increased TGF-β1 is believed to drive the SMAD signalling pathway (26). However, the absence of TGF-β1 in the presence of a significantly activated SMAD pathway does not conform to this understanding.

Several previous studies concluded there was no significant difference between all COPD groups and a control group regarding SMAD levels in small airway tissue (27, 39). These studies, however, did not exclusively use normal non-smoker subjects as the control group. Instead, combine normal lung function smokers or non-smokers with ex-smokers together as a control group. Our results have shown that adding either or both normal lung function smokers and ex-smokers to the control group cohort is not suitable for a control group for these studies. Our findings support the reporting of no significant difference in the levels of TGF-β or SMAD activity between normal lung function smokers and COPD current smokers but showed a highly significant difference between non-smokers and smokers. This indicates that SMAD levels increase in the presence of smoking but could be independent of COPD pathogenesis, and therefore, a baseline of non-smokers must be established for comparison.

It was noted that while normal controls showed significantly higher TGF-β1 in epithelial cells, the location of TGF-β1 was predominately on the apical surface of the cell bound to the cilia as opposed to NLFS and COPD, which showed significantly less TGF-β1, however it was located intracellularly in the apical border of the nucleus or somewhat in the cytoplasm. In normal conditions, TGF-β synthesis and expression are widespread. However, it is stored in a latent form adjacent to the secretory cell (41). Activation of TGF-β receptors through TGF-β1 binding is localised to where TGF-β is released from latency (41). The micrograph of TGF-β1 in NLFS suggests that TGF-β is active intracellularly or being modified in the Golgi complex for secretion (42). This suggests that the TGF-β present in NLFS may be more active in terms of translocation and post-transcription modification and that the TGF-β found in higher amounts in the normal subjects may be latent. The half-life of active TGF-β1 is approximately 2 minutes, compared to 90 minutes in its latent state (43). Binding of active TGF-β1 to the serine-threonine kinase TGF-β type I receptor (TβRI) recruits a constitutively phosphorylated TGF-β type II receptor (TβRII), in turn, phosphorylates the regulatory segment of the TβRI forming a heterotetrameric receptor complex (43). This complex activates the SMAD pathway via receptor complex phosphorylation of the SMADs (43). Some studies have demonstrated a constitutive increased SMAD3 phosphorylation in systemic sclerosis fibroblasts (44). The systemic sclerosis fibroblasts have been shown to be active in organ fibrosis of patients with systemic sclerosis fibrosis, including the lungs (45). It is possible that while the activity of TGF-β1 may diminish, it could still drive SMAD2/3 activation in a constitutive fashion. Similarly, under chronic smoking stimulation, TGF-β type I receptor could be activated constitutively by TGF-β1, which then degrades. However, there is as yet no research exploring the possibility of TGF-β receptors constitutive phosphorylating SMAD2/3 in the absence of TGF-β1, and previous research in large airways demonstrated immunohistochemically detectible increases in TGF-β1 expression in COPD patient utilising the same methodology (26). However, this may be possible to replicate in an *in vitro* or *in vivo* TGF-β knock-out model.

Previous studies have demonstrated a link between EMT and COPD changes in large airways (18). These studies concluded that the EMT pathway in large airways showed classical features of EMT type 3, linked with cancer development and metastasis (19, 46). Cancer formation in large airways is common, especially squamous cell carcinoma (46). EMT type 2 is seen in wound healing, tissue regeneration and fibrosis through the action of recruited myofibroblasts and is the likely EMT pathway at the core of small airway fibrosis and obliteration (18, 19, 21, 37). In this study, we used the EMT markers to indicate EMT progress in the small airways of COPD patients. Vimentin and S100A4 are the core markers for mesenchymal transition-mediated fibrosis, which exhibit EMT type 2 activities. Extensive work has been carried out investigating the drivers of EMT type 3, however, this study suggests that EMT type 2 in small airways is driven by SMAD signalling independent of TGF-β control.

Independently of TGF-β1 activity, transcription factor pSMAD2/3 epithelial levels in the three test groups were significantly higher compared to NC. This increase of pSMAD2/3 was initiated by smoking, indicating that increased epithelial pSMAD2/3 level is stimulated by smoking in the absence of a COPD phenotype. An additional increase was noted in current smokers with COPD, indicating an exacerbation of pSMAD2/3 expression in COPD. Patients with COPD that ceased smoking saw only a small reduction of pSMAD2/3 in the basal cells. Inhibitory SMAD7 also showed a significant increase in the epithelium of all test groups compared to the normal controls, with no significant difference between the test groups. However, unlike SMAD2/3, there was no significant reduction in SMAD7 in COPD patients who no longer smoked.

The proportion of epithelial pSMAD2/3 to SMAD7 demonstrates that although both biomarker levels increased in NLFS and COPD, there was a significantly disproportionate increase in SMAD7 compared to pSMAD2/3 in comparison to the normal ratio. This disproportionate ratio could explain the continued increase in basal cell SMAD7 despite the decreased basal cell pSMAD2/3 even after smoking cessation. As SMAD7 is an inhibitor of SMAD2/3 activation and, ultimately, the SMAD signalling pathway, levels of SMAD 7 would continue to be elevated to establish a normal ratio and balance the disproportionate levels of SMAD2/3. In normal conditions, basal cells act as progenitors’ cells that can differentiate in a variety of cell lines (47). In smokers, basal cells are the first cell to undergo histological changes despite not being the most apical cell (48). This sees proliferation and altered phenotypes (48). It, therefore, can be hypothesised that on smoking cessation, the proliferative and adaptive basal cells would also be the first to return to a normalised level, as seen with pSMAD2/3. This is further corroborated by the decrease in the total number of basal cells staining positive for SMAD7/pSMAD2/3. This shows that smoking, in the absence of COPD, increases both SMAD7 and pSMAD2/3 with a disproportionately higher increase in SMAD7. Upon smoking cessation, the proportions of SMAD7/pSMAD2/3 revert to pre-COPD levels, as seen in NLFS, and the total number of cells staining decreases from approximately 90 basal cells to 70 cells per mm of RBM. This suggests that some changes that occur due to smoking resulting in increased SMAD are stabilised through COPD development, making them irreversible even following smoking cessation.

It has been well established that hundreds of genes demonstrate either increased or decreased expression as a direct result of smoking, irrespective of COPD development (48). As phosphorylated SMAD2/3 forms a receptor complex that interacts with SMAD4 for translocation to the nucleus (26, 27), the nuclear location of pSMAD2/3 demonstrates active translocation. The nuclear location of SMAD2/3 in all test groups indicates active transcription independent of TGF-β1 (**Figure 1**). Active pSMAD2/3 nuclear translocation and EMT-related gene activation are further supported by increased pSMAD2/3 positive cells in the RBM. Histologically normal small airways show a very thin RBM that is acellular. However, a key histological characteristic of COPD is RBM fragmentation with cleft formation (49). In conjunction with RBM fragmentation, studies have also shown cellular movement across the RBM in conjunction with COPD and smoking (50). This study observed a correlation between the number of migrating cells undergoing EMT in the RBM and lung function. Increased cellular presence in the RBM (indicative of airway remodeling and a COPD phenotype) lung function is reduced as a result of airway wall thickening. This was demonstrated in the significant correlation between pSMAD2/3 positive staining cells in the RBM and basal layer and FEF _25-75%_. FEF _25–75%_. (a marker of early airway obstruction) (51) and defined as the forced expiratory flow over the middle half of the FVC; the average flow from 25 per cent of FVC exhalation to 75 per cent of FVC exhalation (52).

The lack of correlations between SMAD pathway activation in the total epithelium and lung function is unsurprising. While there is a suggested general negative trend, as this paper has demonstrated, the activation of the SMAD pathway in the epithelium is independent of COPD development. Significant increases in epithelial SMAD activation are seen before any impact on lung function.

The primary strength of this study is that we directly investigated human tissue from COPD affected individuals and compared these to a non-smoking control group. Participants’ numbers provided robust data with a good spread of airflow obstruction in small airways. The major limitation of the study is that mechanistic work is needed to derive informative correlations between TGF-β1, SMAD activity and EMT. We plan to do this in future studies.

COPD is a major burden on both individuals and the health sector. EMT has been identified as a driving factor for airway remodeling, and TGF-β1 has been demonstrated to drive EMT type 3 in large airways. This study has shown that the SMAD signalling pathway is not only active independently of TGFβ-1, but TGFβ-1 is decreased in COPD patients and normal lung function smokers. It should also be noted in this context that due to unavailability of lavage samples we were unable to detect TGF-β1 or SMAD proteins in lavage fluid, which could have further provided information regarding TGF-β1 dependent or independent SMAD signalling. While future work is indeed needed to identify the activator of the SMAD signalling pathway, this study supports a deviation from the classical TGF-β drive EMT type 3 and suggests different factors that drive small airway fibrosis and EMT type 2.

## Data availability statement

The raw data supporting the conclusions of this article will be made available by the authors, without undue reservation.

## Ethics statement

Tasmania Health & Medical Human Research Ethics Committee approved the study (H0012374). The non-smoking normal control tissues were obtained through the James Hogg Lung Registry, the University of British Columbia, with approval from the Providence Health Care Research Ethics Board H00–50110. The patients/participants provided their written informed consent to participate in this study.

## Author contributions

SB wrote the first draft of the manuscript, performed experiments and analysis of data, microscopic quantification of tissue changes, and prepared figures. WL edited the manuscript, performed experiments, and assisted in the analysis of data and interpretation. CC, GH, and JL edited and revised the manuscript. AH provided clinical samples. GS and TH provided clinical samples, and edited, and revised the manuscript. ME edited the manuscript, analyzed data, and interpreted the results of experiments, assisted in designing experiments. SS conceived and designed research, edited and revised the manuscript, interpreted results of experiments, designing experiments and data analysis, supervision, and provided funding. All authors contributed to the article and approved the submitted version.
